# Eco-Structured Adsorptive Removal of Tigecycline from Wastewater: Date Pits’ Biochar versus the Magnetic Biochar

**DOI:** 10.3390/nano11010030

**Published:** 2020-12-24

**Authors:** Marwa El-Azazy, Ahmed S. El-Shafie, Saeed Al-Meer, Khalid A. Al-Saad

**Affiliations:** Department of Chemistry and Earth Sciences, College of Arts and Sciences, Qatar University, Doha 2713, Qatar; aelshafie@qu.edu.qa (A.S.E.-S.); salmeer@qu.edu.qa (S.A.-M.); kalsaad@qu.edu.qa (K.A.A.-S.)

**Keywords:** tigecycline, antibiotics, removal, wastewater, adsorption, date pits, magnetic biochar

## Abstract

Non-magnetic and magnetic low-cost biochar (BC) from date pits (DP) were applied to remove tigecycline (TIGC) from TIGC-artificially contaminated water samples. Pristine biochar from DP (BCDP) and magnetite-decorated biochar (MBC-DP) were therefore prepared. Morphologies and surface chemistries of BCDP and MBC-DP were explored using FT-IR, Raman, SEM, EDX, TEM, and BET analyses. The obtained IR and Raman spectra confirmed the presence of magnetite on the surface of the MBC-DP. SEM results showed mesoporous surface for both adsorbents. BET analysis indicated higher amount of mesopores in MBC-DP. Box–Behnken (BB) design was utilized to optimize the treatment variables (pH, dose of the adsorbent (AD), concentration of TIGC [TIGC], and the contact time (CT)) and maximize the adsorptive power of both adsorbents. Higher % removal (%R), hitting 99.91%, was observed using MBC-DP compared to BCDP (77.31%). Maximum removal of TIGC (99.91%) was obtained using 120 mg/15 mL of MBC-DP for 10 min at pH 10. Equilibrium studies showed that Langmuir and Freundlich isotherms could best describe the adsorption of TIGC onto BCDP and MBC-DP, respectively, with a maximum adsorption capacity (*q_max_*) of 57.14 mg/g using MBC-DP. Kinetics investigation showed that adsorption of TIGC onto both adsorbents could be best-fitted to a pseudo-second-order (PSO) model.

## 1. Introduction

With the increased recognition of human health and the consequent progress in healthcare, pharmaceutically active materials (PhAMs) are becoming a core part of the everyday routine. As a terminology, PhAMs comprises drugs (with their different structures, therapeutic categories, and formulations), personal care products, X-ray contrast media, etc. [1,2]. Reaching natural water from diverse sources (e.g., manufacturing sites, humans’ and animals’ discharge, run-offs from hospitals, etc.), PhAMs are increasingly detected in wastewater. Reported concentrations and even being at the subclinical levels represent a risk, not only for the ecosystem, but for the health of the aquatic microorganisms, humans, and animals as well [3,4,5].

Antibiotics represent an enormous category of PhAMs that is globally used for treatment and control of infectious diseases. Tetracyclines (TCs) are broad spectrum antibiotics that share a common basic structure of four linearly fused rings to which a variety of functionalities are attached [6,7]. TCs are among the most used antibiotics both within therapeutic and veterinary rehearsals. For veterinary purposes only, 2500 tons are consumed annually in Europe [8]. As per the reported risk quotient (RQ) [3], tetracycline (the parent drug) is among the 14 pharmaceuticals posing a high risk to the environment. Reported concentrations of TCs in wastewater were in the range of 0.1–1.0 ppb [9]. The high risk of TCs and antibiotics in general stems from the consequences of its administration at a sub-lethal concentration, where new species that are antibiotic-resistant have emerged [10]. Moreover, the presence of even traces of TCs in drinking and wastewater would raise several concerns about both the proficiency of wastewater treatment plants (WWTPs) as well as the instigated remediation techniques [11].

**Figure 1:**
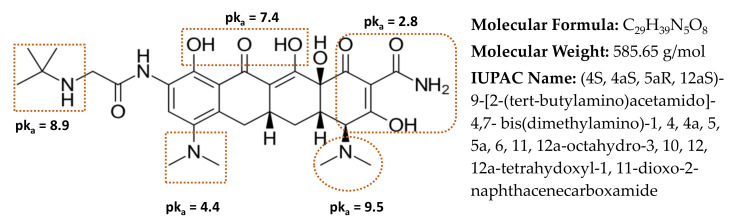
Chemical structure of tigecycline (TIGC) together with the relevant data from [12,13].

Tigecycline (TIGC), the most recent member of the TCs family, belongs to the third generation, Figure 1 [12,13]. TIGC was approved by the US Food and Drug Administration (FDA) on 2005 and was on the list of essential medicines of the World Health Organization (WHO) until 2019. Approval of TIGC came up as a response for the escalating rate of antibiotic resistance [14,15]. TIGC is of low toxicity compared to the other TCs and commonly used in the treatment of both Gram-positive and Gram-negative bacteria, comprising those of multi-drug resilience. Therefore, cases of MRSA (methicillin-resistant Staphylococcus aureus) such as complicated skin and intra-abdominal infections are currently treated with TIGC [16]. Nonetheless, administration of TIGC was associated with cases of unknown deaths, an issue that pushed the FDA to issue a black box warning for TIGC in 2010 [17]. Like other members of the family, TIGC reaches the aquatic environment through the same previously mentioned routes. Yet, with the emergence of *Tet(X)* gene, which is widely available in aquatic environments and wastewater treatment systems, the situation is worsened. This gene could degrade the last-resort TIGC drug, causing increased microbial resistance [18,19].

These apprehensions were motivating to develop a novel approach for the removal of TIGC from contaminated water samples. A literature survey shows that the removal of the TCs family from wastewater has been reported, utilizing a variety of approaches, e.g., adsorption, photocatalysis, microbial degradation, membrane filtration, electrocatalytic oxidation, etc. [19,20,21,22,23,24,25]. Yet, most of these reports, if not all, to the best of our knowledge, were focused on the elder members of the family with almost no effort being made for the removal of TIGC. By and large, adsorption was the thematic approach among the reported treatment efforts for TCs. Offering compelling advantages such as excellent removal capability, a high-quality effluent, fast kinetics, simplicity with an easy-to-implement design, selectivity for certain pollutants, and possibility of adsorbent revitalization, adsorption is seen as a *potent* wastewater treatment approach [26].

Yet, adsorption is economically exhausting due to adsorbents’ cost. Moreover, the adsorption process is affected by several variables. Managing these variables is an intricate task, especially if being tackled employing the conventional univariate approach. Trials to overcome these glitches were conducted utilizing two approaches: upcycling of agro-wastes and optimization of process variables exploiting factorial designs. The output of coupling of the former to the latter is a green approach in which resources and method greenness are greatly preserved [27,28,29,30,31,32,33,34,35,36,37,38].

Biochars (BC) derived from agro-wastes have attracted lots of attention recently. With their high surface area, liability for functionalization, low cost, and possibility of regeneration, BC represent ideal adsorbents. Magnetization of the BC offers an extra advantage where the existence of magnetic nanoparticles with their tiny particle size, high surface-area-to-volume ration, rapid removal kinetics, possible recovery, and most significantly magnetism help in developing a unique wastewater treatment system [39,40,41]. Several reports on the utilization of BC (pristine and magnetic “MBC”) in the removal of antibiotics could be found in literature [20,21,38,39].

In this study, biochar of burnt date pits (BCDP), an agro-waste that is abundantly available in Qatar, and its magnetic biochar (MBC-DP) will be used for remediating the TIGC-artificially contaminated water samples. Process variables including pH, adsorbent dose (AD), concentration of TIGC [TIGC], and the contact time (CT) will be optimized using Box–Behnken (BB) design [35,42]. The objective is to achieve the highest %removal (%R) of TIGC and to maximize the adsorption capacity (*q_e_* (mg/g)) of both adsorbents. The adsorptive efficiencies of the two adsorbents will be related to their surface chemistries and morphologies. The adsorption behavior and kinetics will be explored using the suitable models. The novelty of the current approach, therefore, stems from being the first report on using the BC of an agro-waste (DP, magnetic, and non-magnetic) for the removal of TIGC from wastewater. Moreover, pursuing a control on the process variables using a response surface methodological (RSM) approach will be another plus added to the current study.

## 2. Materials and Methods

### 2.1. Materials, Reagents, Equipment, and Software

Analytical grade reagents were used throughout the experiments. All chemicals (TIGC, hydrochloric acid, sodium hydroxide, ferrous ammonium sulfate hexahydrate (Fe(NH_4_)_2_(SO_4_)_2_·6H_2_O), and ammonium iron (III) sulfate dodecahydrate (NH_4_Fe(SO_4_)_2_·12H_2_O) were the products of Sigma–Aldrich (St. Louis, MO, USA). Palm dates were purchased from a local hypermarket in Doha, Qatar. Date pits (DP) were crushed using a Waring commercial blender. A Memmert ULE700 oven (Memmert, GmbH+Co. KG, Schwabach, Germany) and a Thermolyne 48000 furnace (Thermolyne, IA, USA) were used to dry and burn the clean crushed DP.

Stock solution of TIGC (100 mg/L) was made by dissolving the respective amounts of TIGC in deionized water obtained from a Millipore-Q water system. The pH of water, in which BCDP and MBC-DP were suspended, was adjusted to the desired levels, Table 1 using either 0.1M aqueous solution of HCl or NaOH. pH measurements were carried out using a pH meter (Jenway, Cole-Parmer, Stone, Staffordshire, UK). Concentrations of TIGC before and after adsorption were measured using a UV-Vis spectrophotometer (Agilent diode-array, Agilent, Santa Clara, CA, USA) with 10 mm matched quartz cells. Millex syringe filters (nylon, non-sterile, 0.45 µm) were used to separate the supernatant solution.

Investigation of the functionalities on the adsorbents’ surface was performed utilizing Fourier transform infrared spectroscopy (FT-IR, Perkin Elmer, Shelton, CT, USA, USA). Surface morphology was examined using scanning electron microscope (SEM, FEI, Quanta 200, Thermo Fisher Scientific, Waltham, MA, USA). Carbonization of DP following the heat treatment was explored using Raman spectroscopy (Thermo Fisher Scientific, Waltham, MA, USA). Magnetic and non-magnetic BC were examined using a 200-kV accelerating voltage transmission electron microscope (TEM, TECNAI G2 TEM, FEI, Hillsboro, OR, USA) equipped with energy dispersive X-ray (EDX) spectroscopy and high-angle annular dark-field scanning TEM (HAADF-STEM). TEM samples were prepared by dispersing the MBC-DP or the BCDP sorbents in warm water with the aid of ultrasonic mixing for 20 min. Samples were then mounted on a carbon-coated grid. Measurement of the surface area, pore size, and volume was achieved using a Micromeritics ASAP^TM^ 2020 accelerated surface area and porosimetry system (Micromeritics Instrument Corporation, Norcross, GA, USA). Degassing of samples was primarily applied and then N_2_ adsorption–desorption was studied. Based on the N_2_ isotherms collected at 77 K and employing the Brunauer–Emmett–Teller (BET) equation, the surface area was calculated. Pore volume was obtained using the t-plots and the Barrett–Joyner–Halenda (BJH) equations. Minitab^®^19 software (Minitab Inc., State College, PA, USA) was utilized to build the factorial design.

### 2.2. Preparation of the Biochar (BCDP)

The pits’ biochar was prepared following the procedure mentioned by Al-Saad et al. [31]. Briefly, pits were separated from the dates, cleaned, and washed several times using distilled water followed by hot water. DP were then dried at 100 °C for 2 h then at 60 °C for 3 consecutive days. Dried DP were pulverized into a fine powder. A quantity of 10 g of the crushed DP was placed in a clean dry crucible, covered with a crucible lid, and charred in the furnace at 500 °C for 30 min. The crucible was set aside to cool down and the powder was placed in glass containers, sealed, and kept in the desiccator.

### 2.3. Preparation of the Magnetic Biochar (MBC-DP)

An aqueous mixture containing 200 mL of 0.5M Fe(NH_4_)_2_(SO_4_)_2_·6H_2_O and 400 mL of 0.5M NH_4_Fe(SO_4_)_2_·12H_2_O was prepared by pouring Fe^2+^ solution onto Fe^3+^ solution and then the mixture was stirred with a speed of 400 rpm at 60 °C for 1 h. The magnetic biochar (MBC-DP) was then prepared by co-precipitation following the procedure described by Karunanayake et al. [43] with minor modifications. An amount of 10 g of the BCDP was added to the Fe^2+^/Fe^3+^ mixture and the mixture was stirred at 60 °C for 3 h. Aqueous solution of NaOH (4 M) was then added dropwise to the suspension until the pH value of ~12. The resultant suspension from the previous step was left at room temperature and slowly stirred for 30 min. The suspension was then washed with distilled water (ten times) followed by methanol (five times). The MBC-DP was filtered under a vacuum and dried overnight at 50 °C. The resulting MBC-DP was kept in a plastic container for further use.

### 2.4. Sorption Equilibrium and Kinetic Studies

The equilibrium studies for the sorption of TIGC onto both adsorbents BCDP and MBC-DP were performed by preparing a stock solution, 500 ppm, of TIGC. Several dilutions of the stock solution, 5–400 ppm, were prepared in deionized water, and the pH was adjusted to pH 4.00 ± 0.20 for the BCDP and 10.00 ± 0.20 for MBC-DP adsorbents using 0.1 M HCl and 0.1 M NaOH. Equal quantities of each adsorbent (0.100 ± 0.005 g) were added to 15 mL of the previously prepared solutions, and the produced mixtures were shaken using an automatic shaker at 150 rpm for an equilibrium time of 20 h, followed by filtration using syringe filters. Absorbance of the filtrate was measured at 375 nm. On the other hand, the kinetic studies were performed by mixing 200 mL of the TIGC solution (200 ppm, pH 4.00 ± 0.20 for BCDP, and 10.00 ± 0.20 for MBC-DP) with ~1.0 g of both adsorbents with shaking. Then, an aliquot of 10 mL was taken over a range of 90 min (2, 4, 6, 8, 10, 15, 30, 45, 60, and 90 min), filtered, and the absorbance of the filtrate was measured at the same wavelength, 375 nm.

### 2.5. Box–Behnken (BB) Design

In the current investigation, adsorption of TIGC onto BCDP and MBC-DP was investigated. Box–Behnken (BB) design was the RSM of choice for maximizing the removal efficiency of the tested adsorbents. Four independent variables ([TIGC], CT, pH, and AD) were varied as per the scenario exhibited in Table 2. The design pattern involved 27 basic runs (including three central points, Ct Pt) in one replicate and as one block. The factorial boundaries were selected carefully in order to get the maximum responses. Evaluation of the adsorptive power of the two adsorbents was accomplished utilizing two quantities: %R and *q_e_*. These quantities were calculated using Equations (1) and (2), respectively, and the obtained values are listed in Table 2. Predicted values were computed using Minitab^®^19, Table 2.

(1) $$\left(\%R\right)=\;\frac{C_{0}-C_{e}}{C_{0}}\;\times \;100\%$$

(2) $$\left(q_{e}\right)=\;\frac{C_{0}-C_{e}}{W}\;V$$

where C_0_ (mg/L) signifies the initial concentration of [TIGC] solution, C_e_ is the concentration of the [TIGC] solution at equilibrium, V stands for the volume of the solution (L), and W is the weight of the adsorbent used (g).

## 3. Results and Discussion

### 3.1. Adsorbents’ Characterization

#### 3.1.1. FT-IR and Raman Spectroscopic Analyses

**Figure 2:**
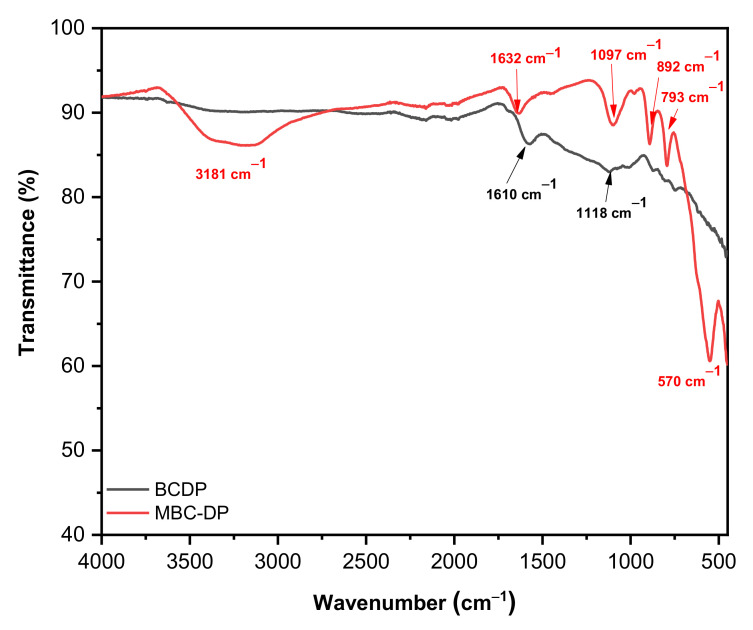
FT-IR spectra of BCDP and MBC-DP.

Being rich in functionalities on their surfaces, agro-wastes can scavenge the pollutants efficiently. Pyrolysis of the lignocellulosic biomasses could destroy and eliminate the organic matter and expose moieties such as −OH, C=O, C=C [31,44]. In the current investigation, exploration of the existence of functional groups on the adsorbents’ surface was performed using FT-IR analysis. Figure 2 shows the FT-IR spectra for both adsorbents. As shown, a sharp absorption peak could be observed at 570 cm^−1^ in case of MBC-DP (absent in case of BCDP). This peak could be attributed to the Fe–O bond vibration and is characteristic for the Fe_3_O_4_-magnetic nanoparticles implying the presence magnetite on the surface of the biochar [31,45,46,47,48]. Moreover, an absorption band at 3181 cm^−1^ in case of MBC-DP could be assigned to the hydroxyl (–OH) stretching vibration stemming from adsorption of atmospheric water or probably from the alcohol used in the washing process. On the other hand, several common peaks appear in the spectra of both samples. For example, the absorption band at 1610 and 1632 cm^−1^ in BCDP and MBC-DP, respectively, might be corresponding to the N–H bending vibration of the quinolines moiety. In addition, the absorption peak at 1118 cm^−1^ in BCDP, and 1097 cm^−1^ in MBC-DP could be attributed to the C–O stretching of aliphatic ether. Additionally, the peaks at 892 and 793 cm^−1^ might be attributed to the C=C bending of the alkene. The obtained data show the existence of several functional groups on the surface of both adsorbents, an issue that might have a significant effect on their adsorption efficiency.

**Figure 3:**
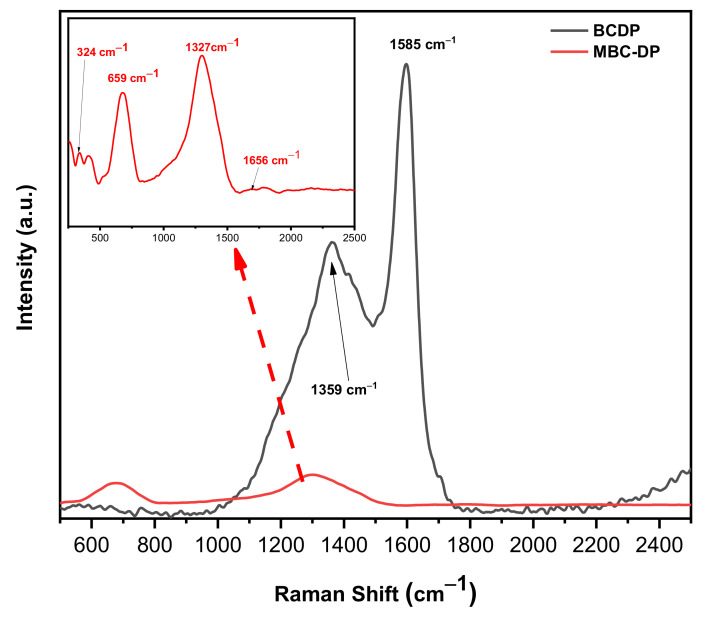
Raman spectra of the as-prepared adsorbents, BCDP and MBC-DP.

Figure 3 shows the Raman spectra of both adsorbents. As could be observed, the spectrum of BCDP shows two strong peaks at 1351 and 1585 cm^−1^, which correspond to the D- and G-bands. These two peaks are unique for carbonaceous materials. In general, the band near 1350 cm^−1^ could be assigned to the sp^3^-bonded (tetrahedral) carbons, while the band near 1500 cm^−1^ could be attributed to the sp^2^-bonded heteroatoms carbons [49]. The MBC-DP spectrum shows two weak broad peaks centered at 324 and 659 cm^−1^, which could be associated with the Fe–O bonds in magnetite [50,51,52].

Furthermore, one intense peak at 1327 cm^−1^ and another weak peak at 1656 cm^−1^ were observed, which could be related to the biochar material. The obtained Raman data and the IR spectrum clearly show the presence of magnetite on the surface of the biochar.

#### 3.1.2. SEM, EDX, and TEM Analyses

**Figure 4:**
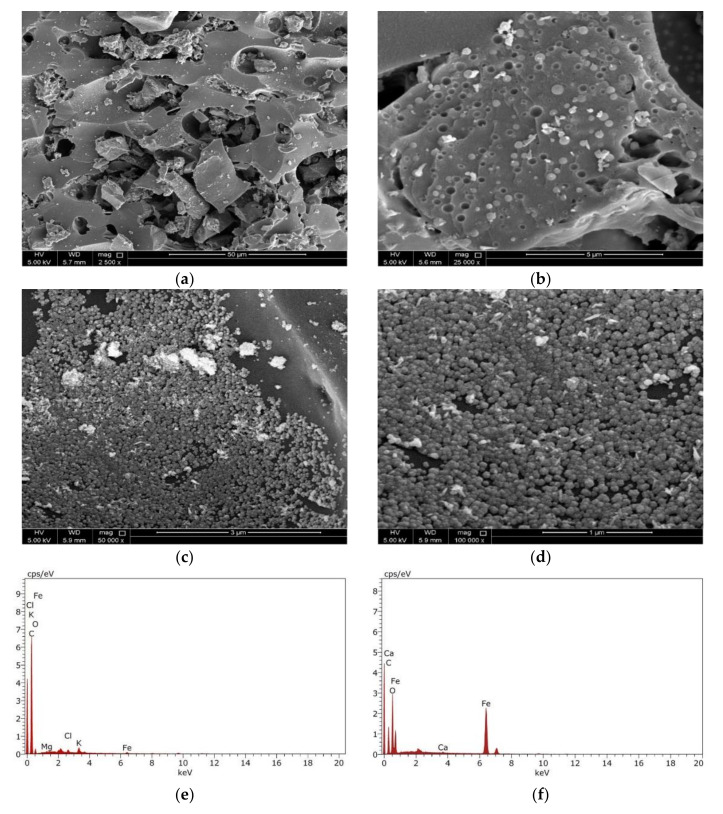
SEM micrographs of BCDP with magnification (**a**) 2500×, (**b**) 25,000×, and MBC-DP with magnification (**c**) 50,000×, (**d**) 100,000×, and EDX analysis for BCDP (**e**) and MBC-DP (**f**).

The morphology of the adsorbent’s surface plays an essential role in controlling the adsorption capability of the adsorbent. The surface morphology characteristics were investigated using SEM together with the TEM analyses. The data shown in Figure 4a,b display the BCDP’s SEM micrographs before loading of the magnetite at different magnifications. As could be observed from these micrographs, the surface of BCDP is porous with mainly mesopores, which will be confirmed later by the BET analysis.

On the contrary, and following the loading of magnetite, the surface of MBC-DP (Figure 4c,d) shows the presence of magnetite nanoparticles. This finding was further confirmed using the EDX analysis shown in Figure 4d,f. EDX analysis shows that the BCDP consists mainly carbon (87%) and oxygen (11%), with trace amounts of other elements including (Mg, Ca, and K). On the other hand, EDX analysis of MBC-DP shows that the concentration of carbon has decreased to 50%, while the iron concentration has increased to 20%, and oxygen to 29%. Presence of iron and oxygen at higher concentrations would prove the formation of iron oxide in the presence of carbon as a supporting material.

**Figure 5:**
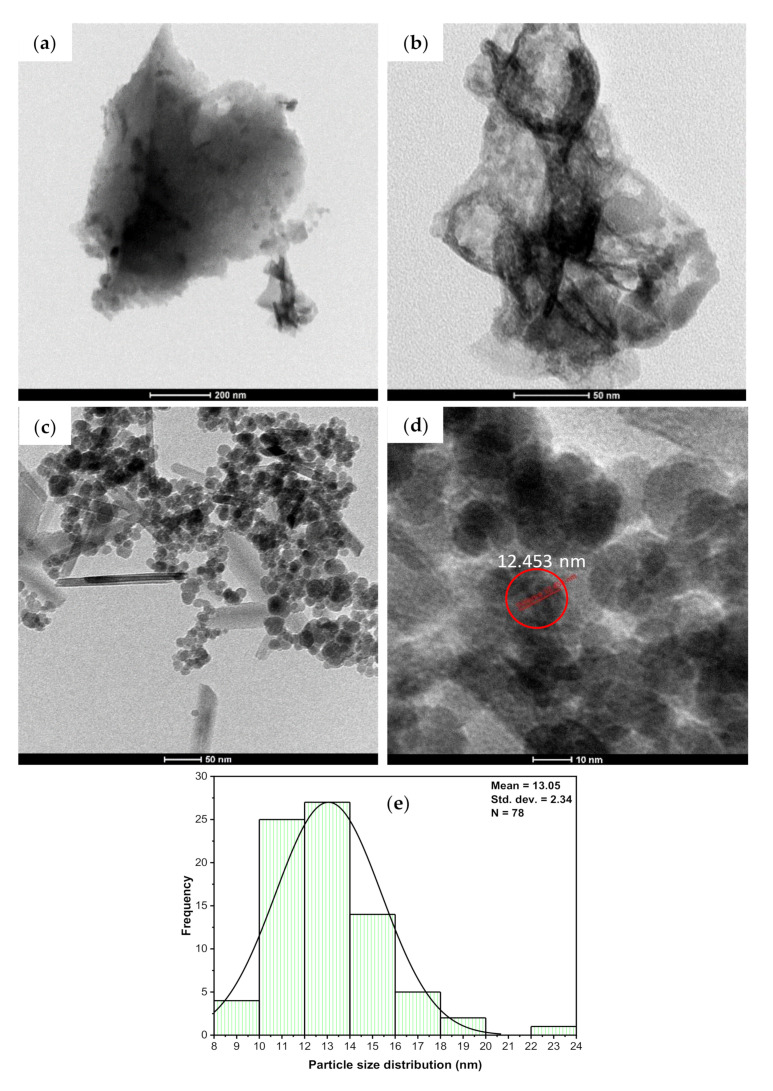
TEM images of BCDP with a scale of (**a**) 200 nm, (**b**) 50 nm, and MBC-DP with a scale of (**c**) 50 nm, (**d**) 10 nm, and (**e**) PSD results for MBC-DP.

Using TEM analysis, microstructural characterization of nanoparticles on the surface of MBC-DP was performed, and the results are illustrated in Figure 5. In agreement with the captured SEM micrographs, the obtained TEM images for BCDP showed a clear surface without any particles, Figure 5a,b. On the other hand, the obtained data in Figure 5c,d show uniform spherical shape nanoparticles. Figure 5e shows the particle size distribution (PSD) of MBC-DP, with a particle size range of 10–20 nm. As also can be perceived from the figure, more than 75% of the particles’ size is approximately 13.05 ± 2.34 nm, confirming that the prepared nanoparticles were uniform in size.

Together with the functional groups’ analysis presented in the FT-IR spectrum, the presence of the magnetite nanoparticles would increase the adsorption capacity of the MBC-DP, making it an ideal adsorbent for TIGC as revealed by the high %R that hits 99.91% compared to 77.31% in case of the pristine BCDP [53].

#### 3.1.3. Brunauer–Emmett–Teller (BET) Analysis

**Figure 6:**
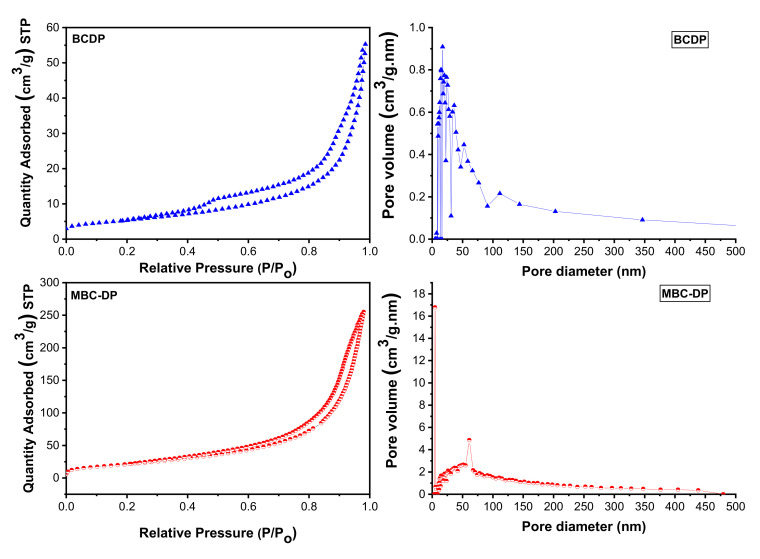
BET analysis of the as-prepared samples of BCDP and MBC-DP.

BET analysis, Figure 6 and Table 3, reveals that the Langmuir surface area of MBC-DP (86.06 m^2^/g) is higher than the surface area of the BCDP (30.45 m^2^/g). This finding might be attributed to the existence of magnetite nanoparticles on the biochar’s surface. By and large, an adsorbent with a smaller particle size could have a larger surface area, an issue that in turn supports an increased uptake of TIGC using MBC-DP. On the other hand, the BCDP samples showed two types of pores; mesopores (2–50 nm) and macropores (higher than 50 nm), compared to the MBC-DP, which showed mainly mesopores and a lower amount of macropores. This could be explained considering the coverage of the pores by the magnetite nanoparticles. The BET adsorption isotherm was of type IV for both adsorbents, implying the occurrence of both monolayer and multilayer adsorption followed by capillary condensation. The hysteresis loop for both samples is H3 type, inferring loose masses of plate-like particles forming slit-like pores [46,54].

### 3.2. Response Surface Methodology (RSM)

Recently, RSM has been widely used for modelling and managing of a variety of wastewater remediation problems. As a symmetrical response surface methodological approach, BB design covers the domain in the central points and hence could evaluate the experimental errors. In other words, BB design entails combinations at the central points of the edges of the process space as well as at the center. Moreover, this design is independent and does not have an engrained full or fractional factorial design points. Added to that, BB is a quadratic design that involves an investigation of each variable at three levels and could be used when number of variables is three or more. Therefore, BB design is usually seen as an economical substitute for the traditional central composite designs that could require more factorial levels [27,28,35,42,55]. In the current investigation, BB design was chosen to optimize the measured responses (%R and *q_e_*) as a function of the four variables. Table 1 shows the investigated variables and their three levels.

### 3.3. Response Modelling and Statistical Analysis

**Figure 7:**
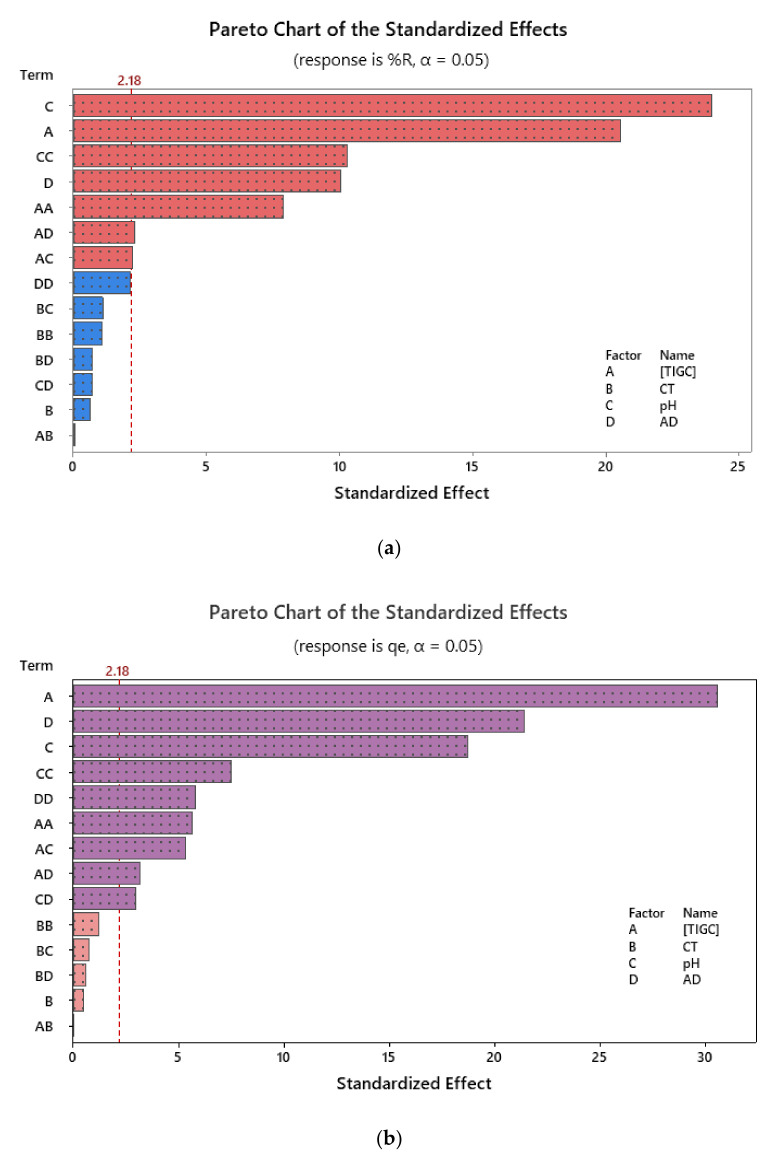
Pareto chart of standardized effects using MBC-DP as adsorbent where (**a**) %R; (**b**) *q_e_* (mg/g) are the measured responses, respectively. Data were obtained following response transformation.

The effectiveness of the operational parameters on the adsorption of TIGC onto both BCDP and MBC-DP and their statistical significance was studied, and the experiments were conducted following the scenario shown in Table 2. The impact of the tested variables on the measured responses was visualized using charts such as Pareto chart of standardized effects. Figure 7 shows an example for the effect of the four variables, two-way and quadratic interactions on both %R and *q_e_* using MBC-DP as adsorbent. As could be figured out from the shown charts, pH was the most influencing variable in case of %R, compared to [TIGC] in case of *q_e_*. Using BCDP as adsorbent, [TIGC] was the most effective variable in both cases—Figures are not shown.

The output of using factorial designs is usually a mathematical model that describes the relationship between independent and dependent variables. This model usually portrays the influence of the independent variables in terms of direction (sign) and magnitude (coefficient) of the impact. Moreover, such a model can be applied to predict the response for given levels of each variable. Equations (3)–(6) show the resultant models in terms of the uncoded variables with respect to the measured responses:

√%R_(BCDP)_ = 1.893 + 0.14734 [TIGC] − 0.01939 CT − 0.3688 pH + 0.02718 AD − 0.000532 [TIGC]^2^ + 0.000154 CT^2^ + 0.03099 pH^2^ − 0.000107 [TIGC] × CT − 0.003643 [TIGC] × pH − 0.000549 [TIGC] × AD + 0.001939 CT × pH + 0.001994 pH × AD,(3)

√*q_e_*_(BCDP)_ = 1.429 + 0.08178 [TIGC] − 0.00511 CT − 0.2066 pH − 0.02438 AD − 0.000223 [TIGC]^2^ + 0.000049 CT^2^ + 0.01328 pH^2^ + 0.000177 AD^2^ − 0.000035 [TIGC] × CT − 0.000499 [TIGC] × pH − 0.000356 [TIGC] × AD + 0.000774 CT × pH − 0.000027 CT × AD + 0.000470 pH × AD,(4)

√%R _(MBC-DP)_ = 0.93 − 0.09407 [TIGC] + 0.0049 CT + 1.985 pH + 0.0212 AD + 0.000380 [TIGC]^2^ + 0.000066 CT^2^ − 0.1113 pH^2^ − 0.000104 AD^2^ + 0.000004 [TIGC] × CT + 0.001835 [TIGC] × pH + 0.000128 [TIGC] × AD − 0.001038 CT × pH − 0.000045 CT × AD + 0.000580 pH × AD,(5)

√ *q_e_*_(MBC-DP)_ = 0.573 + 0.02624 [TIGC] + 0.00055 CT + 0.7104 pH − 0.02061 AD − 0.000127 [TIGC]^2^ + 0.000034 CT^2^ − 0.03805 pH^2^ + 0.000131 AD^2^ − 0.000001 [TIGC] × CT + 0.002082 [TIGC] × pH − 0.000083 [TIGC] × AD − 0.000328 CT × pH − 0.000017 CT × AD − 0.001153 pH × AD,(6)

As could be observed from Equations (3)–(6), the overall effect of any variable on either response could be computed considering the linear, quadratic, and the two-way interactions of that variable, as illustrated by each equation. Model summaries, Table 4, show high value for the coefficient of determination (R^2^) and R^2^-adjusted (R^2^-adj), reflecting the linearity of obtained models. Models’ capability to predict new observations can be foreseen from the high values of R^2^-predicted (R^2^-pred). The difference between the experimental and predicted values was assessed by the values of relative error (RE), Table 2. Obtained RE values were small enough to reflect the absence of difference between both values.

The significance of these mathematical models was further investigated applying the variance analysis (ANOVA) at 95.0 confidence interval (95.0 CI), and the results are presented in Table 5. Displayed data confirm the findings of the Pareto charts as well as the previously revealed mathematical models. As shown in the table, *F*- and *p*-values were used to reflect variables’ significance, where variables with *p*-value less than 0.05 and high *F*-value are recognized as statistically significant and the opposite is true [27]. Lack-of-fit was statistically insignificant implying goodness-of-fit.

### 3.4. Response Optimzation

**Figure 8:**
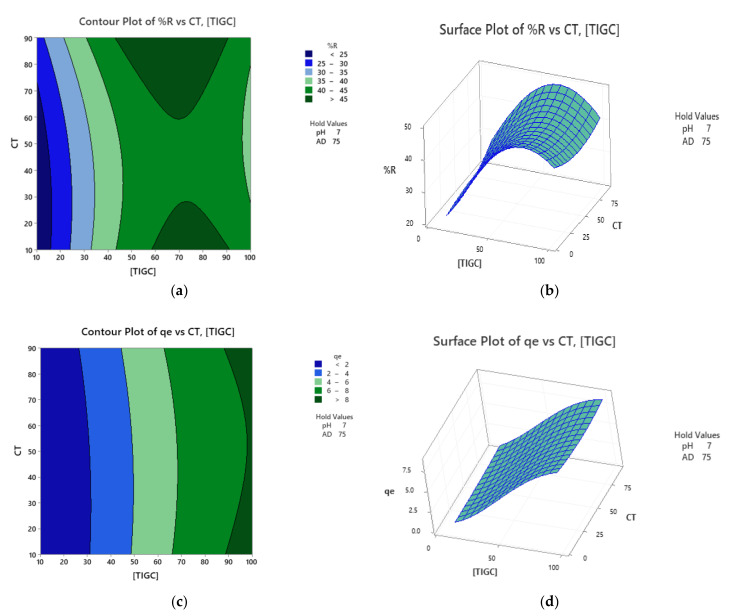
Contour and surface plots using BCDP as adsorbent. (**a**,**b**) %R is the response measured and (**c**,**d**) *q_e_* is the measured response. Data were obtained following response transformation.

Response surface optimization could be performed using a variety of approaches including two- and three-dimensional plots as well as the optimization plots. The two-dimensional (2D) contour plots relate two variables, based on the regression models, to the measured response in the form of contour lines. Surface plots, the three-dimensional (3D) representation, correlate two independent variables (*x-*and *y-*axes) as well as the response surface (*z-*axis) in a 3D format. Figure 8 shows the contour and surface plots for both responses using BCDP as adsorbent. For example, Figure 8a shows a correlation between CT and [TIGC] versus %R surface. The darkest green zone expresses regions in which maximum %R could be attained. Figure 8b shows a surface plot for the same correlation in a 3D format. The elevated ridge represents the maximum %R.

Optimization plots, a tool provided by Minitab^®^, was used to obtain the factorial combinations that maximizes the response. The efficiency of this blend was assessed based on the value of the individual desirability function *(d)*, where the closer the value of *d* to 1.000, the more efficient the blend [56]. Operating the optimization plots, a %R of 77.31% could be obtained for a [TIGC] of 33.63 mg/L at pH 10.0, dose of BCDP = 120 mg/15 mL and for a CT of 90 min with a desirability of 1.000. Similarly, a maximum *q_e_* of 24.21 mg/g could be obtained using a blend of 100 mg/L [TIGC], pH 4.0, CT of 10 min and BCDP dose of 30 mg/15 mL. On the other hand, and using MBC-DP as adsorbent, a desirability of 0.9989 was obtained for a targeted %R of 99.91% using a blend of 25.85 mg/L [TIGC], pH 10.0, MBC-DP dose of 120 mg/15 mL, and for CT of 10 min, while a *q_e_* of 25.48 mg/g could be achieved using 100 mg/L [TIGC], pH 10.0, CT of 10 min, and MBC-DP dose of 30 mg/15 mL.

### 3.5. Equilibrium and Kinetics Studies

Equilibrium studies and the adsorption isotherms are important tools that together with the characterization data helped in comprehending the adsorption process of TIGC onto both adsorbents at the equilibrium phase. Similarly, kinetics’ investigation of time and concentration reliant data was performed, as will be portrayed in the next subsections.

#### 3.5.1. Equilibrium Isotherms

**Figure 9:**
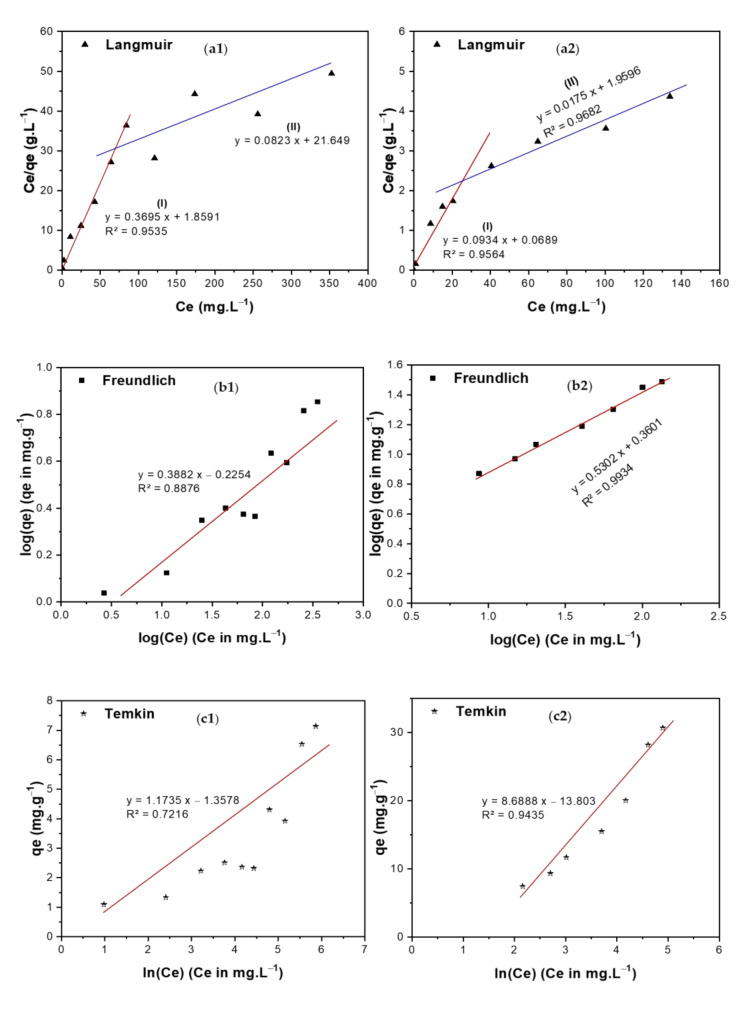

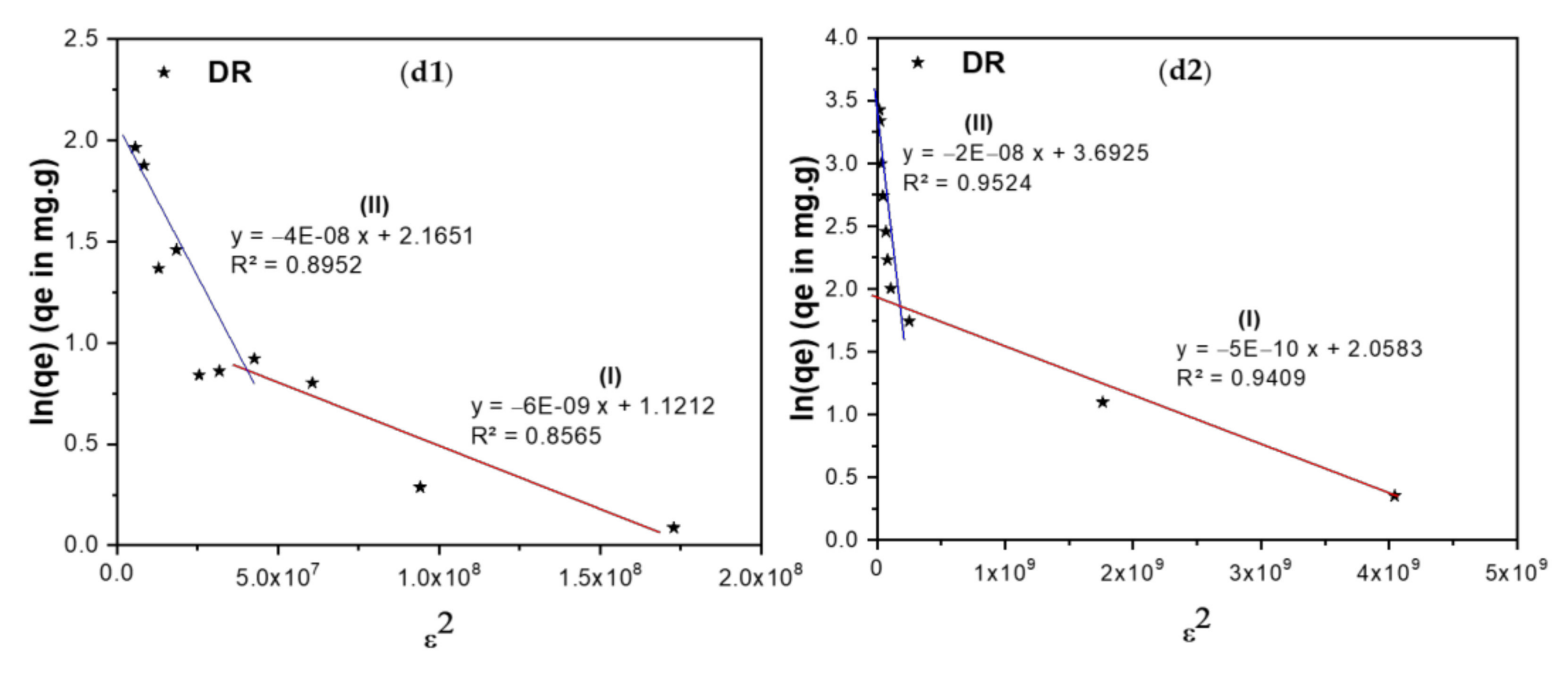
Adsorption isotherms of TIGC onto BCDP and MBC-DP, (**a1**,**a2**) Langmuir, (**b1**,**b2**) Freundlich, (**c1**,**c2**) Temkin, and (**d1**,**d2**) Dubinin–Radushkevich (DR) isotherms.

Adsorption isotherms show the relationship between the concentration of the adsorbate and its extent of accumulation on the adsorbent’s surface at a constant temperature. Langmuir, Freundlich, Temkin, and Dubinin–Radushkevich (DR) isotherms have been used in the current investigation to study the adsorption of TIGC onto the two adsorbents (BCDP and MBC-DP) from an aqueous solution [57,58,59,60], Figure 9(a1–d2).

Langmuir isotherm assumes that the adsorption energy is constant through all sites and that each molecule occupies only one site with no interaction between the molecules. Langmuir isotherm can be represented by Equation (7) and is shown in Figure 9(a1,a2) for both adsorbents.

(7) $$q_{e}=\frac{q_{m}\;K_{L}\;C_{e}}{1+K_{L}\;C_{e}}$$

where *K_L_* is the Langmuir equilibrium coefficient and *q_m_* is the maximum adsorption capacity. Additionally, the Langmuir equation can be represented by using the following dimensionless equation:

(8) $$R_{L}=\frac{1}{1+K_{L}\;C_{0}}$$

In this equation, *C*_0_ (mg/L) denotes the initial concentration, and *R_L_* is the separation factor. According to previous reports, the adsorption favorability can be determined based on the *R_L_*’s value, where if *R_L_* is >1, then the adsorption process is unfavorable. For a value of *R_L_* equal to unity, adsorption is linear. In case the value is between 0–1, adsorption is favorable (occurs spontaneously), and if it equals zero, adsorption is irreversible. The *R_L_* value for both adsorbents was calculated, and it was found to be ˂ 1, signifying that the adsorption process was favorable in both areas I ([TIGC] < 80 ppm) and II ([TIGC] > 80 ppm) and the maximum adsorption (*q_max_*) = 12.15 and 57.14 mg/g for BCDP and MBC-DP, respectively. The value of R^2^ has been used as a basis for deciding the desirability of each fit. In case of BCDP, the R^2^ value for Langmuir isotherm was the highest compared to the other three isotherms implying that Langmuir isotherm can represent the adsorption of TIGC onto BCDP.

Freundlich isotherm is used to describe the heterogeneous surface energies as given by Equation (9):

(9) $$q_{e}=\;K_{F}C_{e}^{\frac{1}{n}}$$

**Figure 10:**
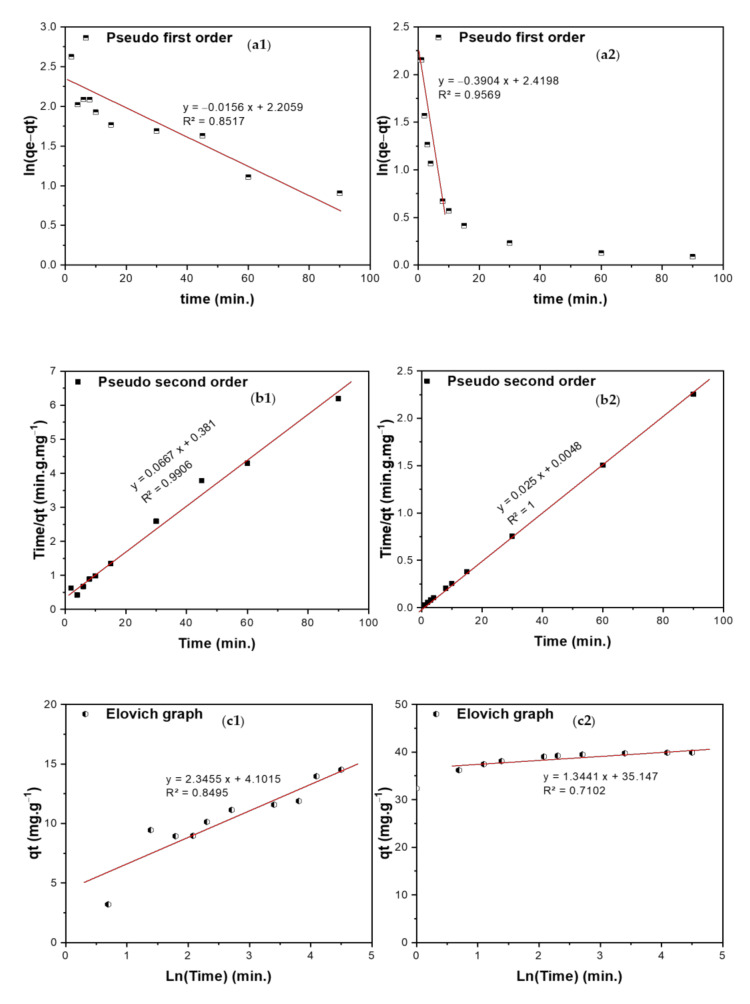

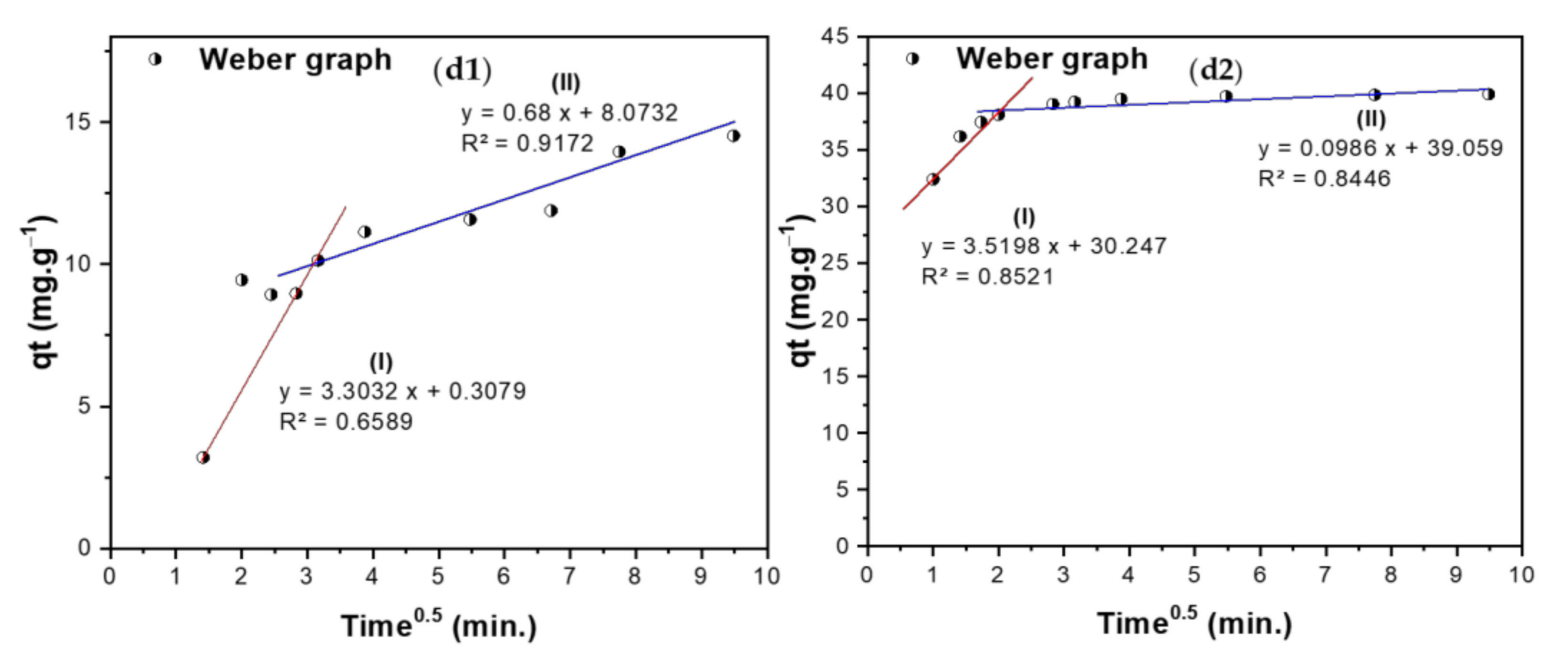
(**a1**,**a2**) Pseudo-first-order, (**b1**,**b2**) Pseudo-second-order, (**c1**,**c2**) Elovich, and (**d1**,**d2**) Intra-particle diffusion (WM) curves of adsorption of TIGC onto BCDP and MBC-DP.

Here, C_e_ is the equilibrium concentration of TIGC (mg/L), *q_e_* is the amount of TIGC adsorbed/unit mass (mg·g^−1^), while *K_F_* (mole·g^−1^) (L·mole^−1^)^1/n^ and 1/*n* are the Freundlich coefficients, Figure 9(b1,b2), Table 6. The Freundlich plot (Figure 9(b1,b2)) demonstrated a good fit with a high R^2^ = 0.9934 confirming that this isotherm is suitable to explain the adsorption of TIGC onto MBC-DP. Additionally, this plot shows that 1/*n* = 0.5302 and *n* = 1.886. The adsorption potential (A = *n*RT) = 4.72 kJ, and hence any TIGC molecule with a potential energy <4.72 kJ, will be adsorbed onto the surface of MBC-DP, and reactions tend to be irreversible and favorable. On the other hand, the BCDP sample, Figure 10(b1,b2), shows that the 1/n value = 0.3882, which is lower than MBC-DP; therefore, *n*= 2.57.

Temkin isotherm (Figure 9(c1,c2)) describes the interaction between the adsorbate and adsorbent; thus, the heat of adsorption of all the molecules in a layer decreases linearly with the adsorbent–adsorbate interactions. The data in Table 6 show that the sorption energy is 280.36 and 2075.8 J/mol for both BCDP and MBC-DP, respectively. This finding cannot determine the type of adsorption. Additionally, the R^2^ value is lower compared to the rest of isotherms, implying that this isotherm cannot describe the adsorption of TIGC onto both adsorbents.

Finally, the DR isotherm shown in Figure 9(d1,d2) reveals the presence of two regions. The first region (region I) appears at very low concentration in which the sorption energy equals 9.13 and 31.54 kJ/mol for both BCDP and MBC-DP, respectively. Adsorption in this region could be chemisorption since the sorption energy is >7 kJ/mol. For region II, at a high concentration of TIGC, the sorption energy was found to be 3.54 and 5.00 kJ/mol for BCDP and MBC-DP, respectively, inferring that the adsorption at a high [TIGC] could be physisorption where the sorption energy is <7 kJ/mol. This finding indicates that the adsorption of TIGC onto both adsorbents goes over two stages; the first stage could be attributed to chemical adsorption to form one layer (confirmed by Langmuir isotherm), and the second is attributed to the physical interaction to form multilayers, and this could interpret findings of Langmuir and Freundlich isotherms. Furthermore, the maximum capacity for MBC-DP in the low concentration region equals 7.83 mg/g, while the maximum capacity in region (II) was 40.15 mg/g, which is almost the same as Langmuir’s maximum capacity.

#### 3.5.2. Kinetic Studies

The kinetics of the adsorption of TIGC onto both BCDP and MBC-DP were investigated using four models: pseudo-first-order (PFO), pseudo-second-order (PSO), Elovich, and Weber–Morris (WM). Figure 10(a1–b2) shows a representation of ln (*q_e_* − *q_t_*) and time/*q_t_* versus time for the PFO and PSO kinetic models, respectively. The calculated parameters of the two models are listed in Table 7. By comparing the R^2^ values, adsorption of TIGC onto both adsorbents could be best described using the PSO model, where the R^2^ = 0.9906 and 1.000 for BCDP and MBC-DP, respectively. Therefore, the adsorption reaction could be represented as follows:

(10) $$TIGC+BCDP\&\;MBC-DP\;\left(\overset{k}{\to }\;\right)\;\left\{TIGC-BCDP\&\;MBC-DP\right\}$$

For the Elovich model given in Figure 10(c1,c2), the initial adsorption was very high in case of MBC-DP, 3.05 × 10^11^ mg·g^−1^·min^−1^ compared to 13.49 mg·g^−1^.min^−1^ in case of BCDP, justifying the superior adsorption efficiency of MBC-DP compared to BCDP. The Weber–Morris (WM) intraparticle diffusion model (Figure 10(d1,d2)) reveals important findings where; besides the intra-particle diffusion, there is another mechanism that controls the diffusion of TIGC. According to the calculated parameters in Table 7, the diffusion occurs over two stages for both adsorbents. In case of BCDP, the adsorption process commenced with a high intraparticle diffusion rate (3.3032 mg·g^−1^·min^−0.5^) and low boundary layer thickness (0.3079 mg/g), then it decreased with time when the boundary layer became 8.07 mg/g. On the other hand, in case of MBC-DP, a different behavior was observed, where the diffusion rate was very high at the beginning of the experiment (3.519 mg·g^−1^·min^−0.5^) and the boundary layer thickness was 30.25 mg/g, implying that the surface is starting to be saturated. Later the diffusion rate decreased with time when the boundary surface became 39.06 mg/g.

### 3.6. Proposed Adsorption Mechanism

Bringing together the characterization, factorial design, kinetics, and equilibrium data, the adsorption mechanism of TIGC onto BCDP and MBC-DP could be portrayed. In other words, adsorption of TIGC onto either adsorbent is affected by a compendium of factors, e.g., existence of functional groups, surface area, particle size, surface charge, aromaticity, pH, and the other statistically significant variables. TIGC, Figure 1, is reported to possess five ionizable groups (two acidic and three basic) [12,13]. The reported pK_a_ values of TIGC were variable and overlapping (2.8, 4.4, 7.4, 8.9, and 9.5), Figure 1. Nonetheless, reports show that TIGC has pK_a_ value of 9.5 (strongest basic) and 2.8 (strongest acidic). Therefore, at a pH value > 9.5, TIGC is expected to exist in the deprotonated form with small amount of the zwitterionic form, while at pH < 2.8, TIGC would be in the protonated form with small amount of the zwitterionic form, and at a 2.8 < pH < 9.5, the three forms of TIGC exist together with the zwitterion form being dominant. On the other hand, BCDP (burnt at 500 °C) has been reported to have a point-of-zero-charge (pH_PZC_) of ~ 6.35 [38,61,62]. Therefore, at high pH values (> 6.35), the adsorbent surface will be negatively charged. In the current investigation, the impact of pH was investigated at three levels: 4.0, 7.0, and 10.0 ± 0.2.

As per the optimization data, the optimum pH value for a maximum %R by BCDP was 10.0 ± 0.2, while the maximum *q_e_* was achieved using a pH value of 4.0 ± 0.2. For both responses, the pH was not the most statistically significant variable, implying that pH does not play the most significant role in adsorption of TIGC onto BCDP compared to the impact of [TIGC], for example. Yet, and together with the data obtained from FT-IR and Raman analyses, at pH 10.0, which is almost equal to the pK_a_ of TIGC (most basic), plausible electrostatic interaction between BCDP (negatively charged) and TIGC (neutral form is dominant but cationic form might also exist) may take place. On the other hand, at pH 4.0, the interaction would occur between the positively charged surface of BCDP and the anionic form of TIGC. Nevertheless, and considering that this mechanism might not be the best-case scenario for the interaction of TIGC and BCDP, the occurrence of π–π electron donor–acceptor (EDA) interaction between the π-system of the BCDP and the aromatic π-system of TIGC might be another possible route [63,64]. It is noteworthy to mention that with increasing the pH, the π-electron density of the TIGC molecule increases and hence the uptake of TIGC.

Using MBC-DP as adsorbent, a pH value of 10.0 ± 0.2 achieved the maximum %R and *q_e_*. It is important to mention that the impact of pH in case of MBC-DP showed a constant plateau in the region of pH 8.0–10.0. Therefore, and in addition to the probability of electrostatic and π–π EDA interactions of TIGC and MBC-DP, the existence of magnetite (rich in O^2-^ around the positively charged iron sites) on the surface of the BCDP that could interact with the cationic form of TIGC represents another mechanism [65]. On the other hand, TIGC, Figure 1, possess several -OH groups and therefore could bond with iron oxide [7]. Moreover, as a magnetic material, iron oxide and through resonance, is capable of changing properties such as surface tension and viscosity of aqueous solutions. Consequently, existence of magnetic nanoparticles on the surface of the BC could improve the mobility of organic contaminants causing their facile adsorption onto the surface of MBC-DP with a probability of contaminant removal using an external magnetic field [66]. Yet, SEM and BET analyses showed that MBC-DP has a higher surface area, pore size, and volume compared to BCDP. These findings might be held accountable for the increased uptake of TIGC onto MBC-DP compared to the pristine BC.

These proposals support the findings of the equilibrium study where chemisorption might be the primary adsorption mechanism; however, physisorption specially at higher concentrations cannot be ruled out.

## 4. Conclusions

Novel and promising adsorbents from biochars of date pits (DP) both non-magnetic (BCDP) and magnetic (MBC-DP) were developed and effectively utilized for the removal of tigecycline (TIGC) from wastewater samples. In this context, both adsorbents were characterized using FT-IR, Raman, SEM, TEM, EDX, and BET analyses. FT-IR and Raman spectra confirmed the presence of magnetite on the surface of the MBC-DP. Brunauer–Emmett–Teller (BET) analysis showed higher amount of mesopores in the prepared MBC-DP compared to non-magnetic BCDP. Controlling of the adsorption process was approached using a multivariate platform, Box–Behnken (BB) design. The target was set to maximize the removal power of both adsorbents, in terms of the percentage removal (%R) and the adsorption capacity (*q_e_*). In this itinerary, MBC-DP was superior to the pristine BCDP with a %R of 99.91% and *q_e_* of 25.48 mg/g. Employing MBC-DP as adsorbent, pH was the most influencing variable in case of %R, compared to the impact of [TIGC] in case of *q_e_*. Nonetheless, [TIGC] was the most effective variable in case of BCDP. Equilibrium isotherms revealed chemisorption and physisorption interactions for both adsorbents at low and high concentrations of TIGC, respectively. According to the Freundlich isotherm, adsorption onto MBC-DP was more favored and irreversible compared to BCDP. A higher adsorption maximum capacity was observed for MBC-DP (*q_max_* = 57.14 mg/g) compared to BCDP (*q_max_ =* 12.15 mg/g). Based on the kinetic studies, pseudo-second-order (PSO) model best fitted the sorption of TIGC onto both adsorbents. The Elovich model showed that the initial adsorption of TIGC was higher using MBC-BDP, 3.05 × 10^11^ mg·g^−1^·min^−1^ compared to 13.49 mg·g^−1^·min^−1^ for BCDP. According to the Weber–Morris (WM) model, the diffusion occurred over two stages for both adsorbents with a high diffusion rate at the first 10 min (>3 mg·g^−1^·min^−0.5^) and a larger boundary layer for MBC-DP (30.25 mg/g) compared to BCDP (0.3079 mg/g).

## Figures and Tables

**Table 1:** Investigated variables at their three levels and the measured responses.

Independent Variables	Code	Units	−1	0	+1
Initial Drug Concentration ([TIGC])	A	mg/L	10.0	55.0	100.0
Contact Time (CT)	B	min	10.0	50.0	90.0
pH	C	pH unit	4.0	7.0	10.0
Adsorbent Dose (AD)	D	mg/15 mL	30.0	75.0	120.0
Responses	Percentage Removal (%R)				
	Adsorption Capacity (*q_e_*, mg/g)				

**Table 2:** Experimental scenario using BCDP and MBC-DP as adsorbents. Measured and predicted values of the two responses are revealed.

Variables					BCDP							MBC-DP				
Trial No	[TIGC]	CT	pH	AD	%R Obs. *	%R Pred. **	RE ***	*q_e_* Obs.*	*q_e_* Pred. **	RE ***	%R Obs. *	%R Pred.**	RE ***	*q_e_* Obs. *	*q_e_* Pred. **	RE ***
01	55 (0)	10 (−)	10 (+)	75 (0)	47.79	49.31	0.03	5.24	5.23	0.00	74.58	72.77	0.02	8.15	8.29	0.02
02	55 (0)	10 (−)	7 (0)	30 (−)	37.32	37.82	0.01	10.00	10.14	0.01	48.08	47.7	0.01	13.13	12.24	0.07
03	55 (0)	90 (+)	7 (0)	30 (−)	40.80	41.5	0.02	11.31	11.44	0.01	50.48	51.15	0.01	13.70	12.88	0.06
04	55 (0)	50 (0)	4 (−)	120 (+)	46.47	45.05	0.03	3.18	3.23	0.01	34.21	31.25	0.09	2.34	2.06	0.14
05	55 (0)	50 (0)	7 (0)	75 (0)	42.45	42.81	0.01	4.64	4.64	0.00	61.11	60.63	0.09	6.67	6.63	0.01
06	100 (+)	50 (0)	7 (0)	120 (+)	30.40	31.42	0.03	3.31	3.19	0.04	59.52	62.77	0.05	7.42	7.26	0.02
07	100 (+)	50 (0)	7 (0)	30 (−)	47.25	46.83	0.01	21.72	21.28	0.02	38.11	37.21	0.026	19.12	18.72	0.02
08	55 (0)	90 (+)	10 (+)	75 (0)	62.01	60.53	0.02	6.81	6.61	0.03	74.19	69.99	0.06	8.14	8.01	0.02
09	55 (0)	90 (+)	4 (−)	75 (0)	45.15	44.19	0.02	4.95	4.97	0.00	29.08	30.23	0.048	3.19	3.14	0.01
10	10 (−)	50 (0)	7 (0)	120 (+)	39.36	39.85	0.01	0.81	0.90	0.10	99.99	101.5	0.01	1.24	1.37	0.09
11	55 (0)	50 (0)	7 (0)	75 (0)	43.83	42.81	0.02	4.76	4.64	0.02	63.87	60.64	0.05	6.96	6.62	0.05
12	100 (+)	50 (0)	4 (−)	75 (0)	44.27	44.38	0.00	8.75	8.7	0.01	20.5	19.48	0.05	4.06	4.22	0.04
13	100 (+)	50 (0)	10 (+)	75 (0)	40.86	40.26	0.01	8.56	8.82	0.03	61.76	64.41	0.04	13.68	14.08	0.03
14	55 (0)	90 (+)	7 (0)	120 (+)	53.71	55.17	0.03	3.67	3.60	0.02	66.87	68.82	0.03	4.57	4.97	0.08
15	10 (−)	50 (0)	10 (+)	75 (0)	33.32	33.79	0.01	0.66	0.68	0.03	99.89	104.0	0.04	1.98	1.78	0.11
16	55 (0)	50 (0)	4 (−)	30 (−)	40.00	39.25	0.02	11.01	10.96	0.00	18.9	19.73	0.04	5.27	5.87	0.10
17	55 (0)	10 (−)	4 (−)	75 (0)	44.73	46.52	0.04	5.18	5.37	0.03	24.17	26.68	0.09	2.66	2.77	0.04
18	55 (0)	10 (−)	7 (0)	120 (+)	53.22	50.92	0.05	3.65	3.60	0.01	69.36	70.10	0.01	4.75	5.10	0.07
19	10 (−)	10 (−)	7 (0)	75 (0)	22.03	21.30	0.03	0.33	0.28	0.18	99.85	99.21	0.01	1.99	2.03	0.02
20	55 (0)	50 (0)	10 (+)	30 (−)	40.64	40.87	0.01	11.27	11.15	0.01	52.35	54.79	0.04	14.06	14.97	0.06
21	55 (0)	50 (0)	10 (+)	120 (+)	63.11	62.69	0.01	4.31	4.32	0.00	81.91	78.57	0.04	5.57	5.10	0.09
22	100 (+)	10 (−)	7 (0)	75 (0)	43.07	42.48	0.01	8.58	8.59	0.00	54.84	52.97	0.03	10.97	10.8	0.01
23	10 (−)	50 (0)	4 (−)	75 (0)	16.54	17.32	0.04	0.33	0.29	0.14	58.6	57.43	0.02	0.72	0.58	0.24
24	55 (0)	50 (0)	7 (0)	75 (0)	41.37	42.81	0.03	4.50	4.63	0.03	57.03	60.64	0.06	6.26	6.62	0.05
25	10 (−)	90 (+)	7 (0)	75 (0)	28.14	28.03	0.00	0.57	0.56	0.02	99.91	100.63	0.01	1.99	2.14	0.07
26	100 (+)	90 (+)	7 (0)	75 (0)	40.97	41.26	0.01	8.19	8.44	0.03	55.28	54.40	0.02	10.92	10.98	0.00
27	10 (−)	50 (0)	7 (0)	30 (−)	10.17	9.64	0.05	0.74	0.79	0.06	90.13	86.25	0.04	4.39	4.56	0.04

* Obs.: observed readings; ** Pred.: predicted readings; *** RE = ǀ (Measured value − Actual value)/Actual value ǀ.

**Table 3:** Brunauer–Emmett–Teller (BET) analysis of BCDP and MBC-DP.

Parameters	BCDP	MBC-DP
Langmuir surface area (SA) (m^2^/g)	30.45	86.06
Total pore volume (cm^3^/g)	0.0337	0.3967
Average pore radius (°A)	36.2	80.0

**Table 4:** Summaries of the regression models.

Response	R^2^%	R^2^–adj %	R^2^–pred %
%R _(BCDP)_	99.44	98.95	97.74
*q_e_*_(BCDP)_	99.92	99.82	99.55
%R _(MBC-DP)_	99.13	98.11	95.48
*q_e_*_(MBC-DP)_	99.39	98.69	96.66

**Table 5:** Analysis of variance (ANOVA) for the transformed responses for both adsorbents.

BCDP										
Response	%R					*q_e_*				
Source	DF *	Adj SS *	Adj MS *	*F*-Value	*p*-Value	DF *	Adj SS *	Adj MS *	F-Value	*p*-Value
Model	12	27.9332	2.32777	205.59	0.000	14	26.4377	1.8884	1036.44	0.000
Linear	4	11.3998	2.84995	251.71	0.000	4	21.4541	5.3635	2943.74	0.000
[TIGC]	1	6.8935	6.89353	608.85	0.000	1	15.5857	15.5857	8554.14	0.000
CT	1	0.2567	0.25669	22.67	0.000	1	0.0291	0.0291	15.98	0.002
pH	1	1.3349	1.33487	117.90	0.000	1	0.0724	0.0724	39.75	0.000
AD	1	2.9147	2.91472	257.43	0.000	1	5.7669	5.7669	3165.11	0.000
Square	3	9.9691	3.32304	293.50	0.000	4	2.8157	0.7039	386.35	0.000
[TIGC]^2^	1	6.9513	6.95132	613.95	0.000	1	1.0900	1.0900	598.21	0.000
CT^2^	1	0.3630	0.36302	32.06	0.000	1	0.0326	0.0326	17.91	0.001
pH^2^	1	0.4666	0.46665	41.22	0.000	1	0.0762	0.0762	41.82	0.000
AD^2^						1	0.6882	0.6882	377.69	0.000
2-Way Interactions	5	6.5643	1.31286	115.95	0.000	6	2.1678	0.3613	198.30	0.000
[TIGC] × CT	1	0.1491	0.14908	13.17	0.003	1	0.0156	0.0156	8.58	0.013
[TIGC] × pH	1	0.9675	0.96753	85.45	0.000	1	0.0182	0.0182	9.97	0.008
[TIGC] × AD	1	4.9413	4.94129	436.42	0.000	1	2.0737	2.0737	1138.12	0.000
CT × pH	1	0.2165	0.21648	19.12	0.001	1	0.0345	0.0345	18.96	0.001
CT × AD						1	0.0097	0.0097	5.33	0.040
pH × AD	1	0.2899	0.28991	25.61	0.000	1	0.0161	0.0161	8.84	0.012
Error	14	0.1585	0.01132			12	0.0219	0.0018		
Lack-of-Fit	12	0.1407	0.01172	1.32	0.511	10	0.0199	0.0020	2.04	0.374
Pure Error	2	0.0178	0.00891			2	0.0020	0.0010		
Total	26	28.0917				26	26.4595			
**MBC-DP**										
**Response**	**%R**					***q_e_***				
Source	DF *	Adj SS *	Adj MS *	*F*-Value	*p*-Value	DF *	Adj SS *	Adj MS *	F-Value	*p*-Value
Model	14	69.3253	4.9518	97.62	0.000	14	21.7529	1.5538	140.51	0.000
Linear	4	55.6423	13.9106	274.25	0.000	4	19.2720	4.8180	435.70	0.000
[TIGC]	1	21.3729	21.3729	421.37	0.000	1	10.3384	10.3384	934.92	0.000
CT	1	0.0213	0.0213	0.42	0.529	1	0.0028	0.0028	0.26	0.622
pH	1	29.1387	29.1387	574.47	0.000	1	3.8713	3.8713	350.09	0.000
AD	1	5.1093	5.1093	100.73	0.000	1	5.0595	5.0595	457.54	0.000
Square	4	13.0565	3.2641	64.35	0.000	4	1.9467	0.4867	44.01	0.000
[TIGC]^2^	1	3.1572	3.1572	62.24	0.000	1	0.3511	0.3511	31.75	0.000
CT^2^	1	0.0589	0.0589	1.16	0.302	1	0.0162	0.0162	1.47	0.249
pH^2^	1	5.3537	5.3537	105.55	0.000	1	0.6255	0.6255	56.57	0.000
AD^2^	1	0.2360	0.2360	4.65	0.052	1	0.3742	0.3742	33.84	0.000
2-Way Interactions	6	0.6265	0.1044	2.06	0.135	6	0.5343	0.0890	8.05	0.001
[TIGC] × CT	1	0.0002	0.0002	0.00	0.954	1	0.0000	0.0000	0.00	0.966
[TIGC] × pH	1	0.2454	0.2454	4.84	0.048	1	0.3160	0.3160	28.57	0.000
[TIGC] × AD	1	0.2683	0.2683	5.29	0.040	1	0.1117	0.1117	10.10	0.008
CT × pH	1	0.0621	0.0621	1.22	0.290	1	0.0062	0.0062	0.56	0.469
CT × AD	1	0.0260	0.0260	0.51	0.488	1	0.0036	0.0036	0.32	0.581
pH × AD	1	0.0245	0.0245	0.48	0.500	1	0.0969	0.0969	8.76	0.012
Error	12	0.6087	0.0507			12	0.1327	0.0111		
Lack-of-Fit	10	0.5105	0.0510	1.04	0.585	10	0.1232	0.0123	2.60	0.310
Pure Error	2	0.0982	0.0491			2	0.0095	0.0047		
Total	26	69.9340				26	21.8856			

* DF is degrees of freedom, SS is sum of squares, and MS is mean of squares.

**Table 6:** General and linearized equations of Langmuir, Freundlich, Temkin, and Dubinin–Radushkevich isotherms. Equations’ parameters are also shown.

Isotherm	Equations (Generalized/Linearized Forms)	Parameters	BCDP		MBC-DP	
			(I) (II)		(I) (II)	
**Langmuir**	$q_{e}=\frac{q_{m}\;K_{L}\;C_{e}}{1+K_{L}\;C_{e}}$ $\frac{C_{e}}{q_{e}}=\frac{1}{q_{m}\;K_{L}}+\frac{C_{e}}{q_{m}}$	*q_m_* (mg/g)	2.71	12.15	10.71	57.14
		*K_L_* (L·mole^−1^)	0.19	0.004	1.35	0.090
		R^2^	0.9535		0.9564	0.9682
**Freundlich**	$q_{e}=K_{F}C_{e}^{\frac{1}{n}}$ $\log\left(q_{e}\right)=\log\left(K_{F}\right)+\left(\frac{1}{n}\right)\log\left(C_{e}\right)$	1/n	0.3882		0.5302	
		*K_F_* (mole/g) (L/mole)^1/n^	0.595		2.29	
		R^2^	0.8876		0.9934	
**Temkin**	$q_{e}=\frac{RT}{b_{T}}\;\ln\left(A_{T}\;C_{e}\right)$ $q_{e}=\frac{RT}{b_{T}}\ln\left(A_{T}\right)+\frac{RT}{b_{T}}\ln\left(C_{e}\right)$	*b_T_* (J/mole)	2075.8		280.36	
		*A_T_* (L/mole)	0.314		0.2042	
		R^2^	0.7216		0.9435	
**DR**	$\ln(q_{e})=\ln\left(q_{m}\right)-\beta \epsilon^{2}$	*β*	6 × 10^−9^	4 × 10^−8^	5 × 10^−10^	2 × 10^−8^
	$\epsilon =RT\left(1+\frac{1}{C_{e}}\right)$	*E* (kJ/mol)	9.13	3.54	31.62	5.00
		*q_m_* (mg/g)	3.07	8.72	7.83	40.15
	E=12β	R^2^	0.8565	0.8952	0.9409	0.9524

**Table 7:** The kinetics study results corresponding to Figure 10.

Models	Parameter	BCDP	MBC-DP		
Pseudo-first order (PFO) $\ln\left(q_{e}-q_{t}\right)=\ln\left(q_{e}\right)-k_{1}t$	K_1_ (min^−1^)	0.0156	0.3904		
	*q_e_* (mg/g)	9.078	11.24		
	R^2^	0.8517	0.9569		
Pseudo-second order (PSO) $\frac{t}{qe}=\;\frac{1}{k_{2}q_{e}^{2}}+\frac{1}{q_{e}}$ t *Where K_2_ is rate constant (g·mg^−1^·min^−1^)*	K_2_ (g·mg^−1^·min^−1^)	0.0117	0.130		
	*q_e_* (mg/g)	14.99	40.00		
	R^2^	0.9906	1.0000		
Elovich equation is $q_{t}=\frac{1}{\beta }\ln\left(\alpha \beta \right)+\frac{1}{\beta }\ln\left(t\right)$ is used to predict the sorption mechanism, where *q_t_* is adsorbed quantity at time t; while α and β are initial sorption concentration rate (mg·g^−1^·min^−1^), and desorption constant (g/mg), respectively.	α	13.49	3.05 × 10^11^		
	β	0.426	0.744		
	R^2^	0.8495	0.7102		
Weber–Morris intraparticle diffusion model is used to study the formed layers around the adsorbent and rate-controlling step, which is expressed as $q_{t}=K_{I}t^{0.5}+C$, where *K_I_* is intraparticle diffusion rate constant (mg·g^−1^·min^−0.5^), and C is the boundary thickness effect.	K_I_	3.303	0.680	3.519	0.0986
	C	0.308	8.073	30.25	39.059
	R^2^	0.6589	0.9172	0.8521	0.8446

## Data Availability

The data presented in this study are available within this article. Further inquiries could be directed to the authors.
